# Draft genome sequence of *Virgibacillus salarius* isolated from the San Juan Hypersaline Lagoon, Chihuahua, Mexico

**DOI:** 10.1128/mra.00267-26

**Published:** 2026-05-18

**Authors:** Alejandro S. Cruz-Soto, Claudia C. Hernández-Peña, Marisela Y. Soto-Padilla, Stephanie Viridiana Laredo-Tiscareño, Rodolfo Gonzalez Peña, Erick de Jesús de Luna-Santillana, Luis Alberto Cira-Chávez, Javier Alfonso Garza-Hernández

**Affiliations:** 1 Departamento de Biotecnología y Ciencias Alimentarias, Instituto Tecnológico de Sonora, Ciudad Obregón, Sonora, México; 2 Instituto de Ciencias Biomédicas, Universidad Autónoma de Ciudad Juárez, Ciudad Juarez, Chihuahua, Mexico; 3 Instituto de Ingeniería y Tecnología, Universidad Autónoma de Ciudad Juárez, Ciudad Juarez, Chihuahua, Mexico; 4 Centro de Investigaciones Regionales “Dr. Hideyo Noguchi”, Universidad Autónoma de Yucatán, Mérida, Yucatán, Mexico; 5 Centro de Biotecnología Genómica del Instituto Politécnico Nacional, Reynosa, Tamaulipas, Mexico; California State University San Marcos, San Marcos, California, USA

**Keywords:** *Virgibacillus salarius*, draft genome, sequencing, Mexico, Chihuahua

## Abstract

*Virgibacillus salarius* is a halophilic bacterium of biotechnological interest due to its salt-tolerant enzymes. Here, we report the draft whole-genome sequence and annotation of a *V. salarius* strain isolated from water collected at the San Juan Hypersaline Lagoon, located in the municipality of Ascension, Chihuahua, Mexico.

## ANNOUNCEMENT

*Virgibacillus salarius* is a gram-positive, endospore-forming, rod-shaped halophilic bacterium originally isolated from Gharsa salt lake (Chott el Gharsa), Tunisia, adapted to saline environments, and capable of growth under high salt concentrations and fluctuating physicochemical conditions (1). This species has attracted biotechnological interest due to its ability to produce salt-tolerant enzymes that remain active and stable under high salinity and alkaline pH conditions (2). Enzymes reported from *V. salarius*, including haloalkaliphilic pectinases and thermo/halo-stable cellulases, highlight its potential for industrial applications such as bioprocessing and biomass conversion (3). Genomic and functional characterization of this species provides insights into halophilic adaptation mechanisms and supports the identification of genetic traits relevant for extremophile-based biotechnological applications (1–3).

*V. salarius* was isolated from 1,000 g of sediment collected using a 50 mL sterile Falcon tubes in August 2022 at the San Juan Hypersaline Lagoon, Ascensión, Chihuahua, Mexico (31°03′28.12″ N; 107°53′28.79″ W; 1,293 masl; electrical conductivity: 16.0–17.0 deciSiemens m⁻¹). Samples were enriched in nutrient broth with 5% NaCl and incubated at 37°C for 21 days at 200 rpm. Aliquots collected at 0, 3, 7, 14, and 21 days were serially diluted, plated on nutrient agar, and incubated at 37°C for 3 days. Genomic DNA from a representative isolate, selected based on consistent colony morphology and growth characteristics, was extracted using the Qiagen Blood and Tissue Kit (Qiagen, Germany, Cat. No. 69506), following the manufacturer’s instructions. DNA concentration was quantified with the Qubit double-stranded DNA High-Sensitivity Assay Kit on a Qubit fluorometer (Thermo Fisher Scientific, MA, USA). Whole-genome sequencing was performed as previously described by Atayde-Torres et al. (4), using 500 ng of genomic DNA. Library preparation was conducted with the Nextera Flex Library Kit, incorporating individual indices for barcoding, following the Illumina reference protocol (document no. 10000000254). Finally, sequencing was performed on an Illumina MiniSeq. Illumina paired-end sequencing (2 × 145 bp) generated 3,008,327 read pairs (6,016,654 reads). Read quality was assessed in Galaxy using FastQC v.0.12.x, and reports were visualized with Falco v1.2.4. Reads were quality-filtered and trimmed with fastp v1.0.1 (default settings) to remove adapter sequences, low-quality bases, and short reads (5). Trimmed reads were assembled *de novo* with Unicycler v0.5.1 in short-read mode (*normal bridging*), which internally uses SPAdes v4.2.0 for assembly graph construction (6). Contigs shorter than 500 bp were excluded from downstream analyses. The draft assembly was polished with Pilon v1.20.1 using trimmed reads mapped back to the assembly to improve consensus accuracy (7), and assembly statistics were computed with QUAST v5.3.0 (8). The final polished draft genome comprised 4,296,102 bp across 259 contigs (≥500 bp), with a GC content of 36.57%, an N50 of 31.8 kb, and a largest contig of 114,016 bp. Genome annotation was performed using Bakta Web v1.11.3 (9), which predicted 4,098 protein-coding sequences, 61 tRNAs, 3 rRNAs, 29 ncRNAs, and 1 tmRNA. The strain was identified as *V. salarius* based on OrthoANIu analysis using the EZBioCloud ANI calculator against reference genome GCA_018139605.1 (ANI = 99.65%) (10). Table 1 summarizes the genes associated with halotolerance and osmoadaptation.

**TABLE 1:** Summary of genes associated with halotolerance and osmoadaptation in *V. salarius*

Category	No. of genes
Ectoine/hydroxyectoine (biosynthesis/transport)	7
Betaine/choline/carnitine (uptake/metabolism)	7
Proline transport	3
Na^+^/H^+^ antiporters	9
K^+^ uptake/homeostasis	4
**Total**	**30**

## Data Availability

The draft genome sequence of *V. salarius* was deposited at GenBank under accession number JBVFKU000000000. The project data are available under BioProject acc. PRJNA1406333 and BioSample acc. SAMN54769905. The raw draft genome data were deposited in the Sequence Read Archive (SRA) under SRA acc. SRR37519191. Bakta annotation outputs and the contig-filtered assembly used for the reported QUAST statistics (contigs ≥500 bp) are available via Zenodo (https://doi.org/10.5281/zenodo.18884046). Ethical approval was not required for this study.
